# Randomized Clinical Trial of the Effectiveness of a Home-Based Advanced Practice Psychiatric Nurse Intervention: Outcomes for Individuals with Serious Mental Illness and HIV

**DOI:** 10.1155/2011/840248

**Published:** 2011-05-17

**Authors:** Nancy P. Hanrahan, Evan Wu, Deena Kelly, Linda H. Aiken, Michael B. Blank

**Affiliations:** ^1^Center for Health Outcomes and Policy Research, School of Nursing, University of Pennsylvania, 418 Curie Boulevard, Philadelphia, PA 19204-4217, USA; ^2^Leonard Davis Institute of Health Economics, University of Pennsylvania, 3641 Locust Walk, Philadelphia, PA 19104-6218, USA; ^3^Robert Wood Johnson Foundation Nurse Faculty Scholars Program, RWJF, P.O. Box 2316, Route 2 and College Road East, Princeton, NJ 08543, USA; ^4^Center for Mental Health Policy, School of Medicine, Department of Psychiatry, University of Pennsylvania, 3535 Market Street 3rd Floor, Philadelphia, PA 19104-2648, USA

## Abstract

Individuals with serious mental illness have greater risk for contracting HIV, multiple morbidities, and die 25 years younger than the general population. This high need and high cost subgroup face unique barriers to accessing required health care in the current health care system. The effectiveness of an advanced practice nurse model of care management was assessed in a four-year random controlled trial. Results are reported in this paper. In a four-year random controlled trial, a total of 238 community-dwelling individuals with HIV and serious mental illness (SMI) were randomly assigned to an intervention group (*n=128*) or to a control group (*n=110*). Over 12 months, the intervention group received care management from advanced practice psychiatric nurse, and the control group received usual care. The intervention group showed significant improvement in depression (*P=.012*) and the physical component of health-related quality of life (*P=.03*) from baseline to 12 months. The advanced practice psychiatric nurse intervention is a model of care that holds promise for a higher quality of care and outcomes for this vulnerable population.

## 1. Introduction

People with serious mental illness (SMI), such as schizophrenia or bipolar disorder, are at increased risk of contracting HIV [1]. Contributing factors include poverty, residing in disadvantaged neighborhoods, high substance use, cognitive impairment, and poor access to health care [2, 3]. Individuals with SMI and HIV have complex care needs. Common treatment regimens for SMI and HIV include large numbers of medications with troublesome side effects and frequent appointments with multiple providers. Navigating the health care system, in which general medical care and mental health care treatment operate in silos, requires a high level of communication and organizational skills, skills that are often compromised in this population [4]. The challenge is to provide resources that connect this population to high-quality care and appropriate services that maintain health and functioning in the face of disease progression and ensure that this care is coordinated across multiple providers.

Research shows that care management models with advanced practice registered nurses (APRNs) as providers improve outcomes for high-risk populations [5–7]. However, the effectiveness of these models has not been studied in the population with SMI and comorbid HIV. In this paper, we report results from a randomized controlled trial that tested a care management intervention delivered by advanced practice psychiatric nurses to improve outcomes for individuals with SMI and HIV.

## 2. Background

Serious mental illness (SMI) indicates significant cognitive, mood, or behavioral symptoms that interfere with an individual's capacity to socialize, plan, organize, and function [8]. Diagnoses most associated with SMI include schizophrenia, bipolar disorders, and major depression. Individuals with SMI have higher prevalence of HIV infection than individuals in the general population [1]. Seroprevalence of HIV infection in the U.S. population is 0.43% [9]. One study of Medicaid beneficiaries with SMI reported the risk of HIV infection at 3.7%, with HIV prevalence among people with schizophrenia at 2.8% and prevalence among those with affective disorders at 4.6% [1]. Cooccurrence of SMI and a substance use disorder triples the risk of HIV infection [1]. 

Recent evidence of poor general health of individuals with SMI adds to the complexity of their health conditions. Research shows that persons with SMI die 25 years earlier than those in the general population [10]. One study showed their average age of death to be 51 years, compared with 76 years for Americans overall [11]. Compared with the general population, persons with SMI are 3.4 times more likely to die from heart disease or diabetes, 3.8 times more likely to die from accidents, 5.0 times more likely to die from respiratory ailments, and 6.6 times more likely to die from pneumonia influenza [12, 13]. These disparities are hypothesized to be related to high rates of undetected and untreated general medical conditions. Additionally a high prevalence of metabolic syndromes and infectious diseases have been associated with persons with SMI [14, 15]. Due to system barriers, this population does not regularly access primary care providers and receive routine screening and treatment for these conditions. 

Fragmented mental health care and physical health care systems exact their toll on this population and use scarce public resources ineffectively, and inefficiently. Systems for the delivery of mental health, substance abuse treatment, and general medical care operate independently, communicate with one another inefficiently and often have different financing arrangements and policies [16]. Research shows that SMI consumers have legitimate concerns that their general medical needs may be dismissed as symptoms of their mental illness [17]. Their physical problems are often missed by medical providers and go untreated [17, 18]. Studies describe long wait times, unsupportive health care staff, disrespectful communication, and even ridicule [19]. Such encounters add to the stigmatization and emotional suffering of this population. Avoiding care or being dismissed when seeking care exacerbates health problems and ultimately adds to costs of health care [12].

The public health stakes are high and the problems have complex physical and psychological dimensions. Innovative solutions are needed that bridge organization and professional silos, improve communication of essential clinical information, and provide care management and social supports to prevent costly relapse and other adverse outcomes in this vulnerable population [18]. One such innovation is to link these individuals to a professional with the knowledge and skills to assess, treat, and manage general medical and mental health problems while ensuring they remain connected with their usual care providers, such as case managers, physicians, and health care systems. APRNs have such specialty education and provide highly skilled care that focuses on general medical, mental health, and substance use issues. Many high-risk populations, such as low-birth weight babies, patients with congestive heart failure, and elders with cognitive impairment, have responded with better outcomes when they received APRN models of care [5, 20, 21]. 

Despite evidence of risk factors associated with SMI and HIV, or the risk factor that SMI itself may pose to contracting and spreading HIV, the effectiveness of APRN interventions has not been rigorously studied in this high-risk population. This paper describes a randomized controlled trial of a community-based intervention provided by APRNs and directed at care coordination and at improving adherence to SMI and HIV treatment regimens. Building sustainable health networks between the client and a community of mental health, substance use, and primary care providers was a key objective. Our hypothesis was that, by the 12-month followup, the patients receiving the home-based APRN intervention would have experienced greater improvements in symptoms and quality of life than the control group. Further, we hypothesized that the outcome response would be associated with an APRN dose level. Specifically we hypothesized that a higher APRN dose would be associated with a reduction in psychiatric symptoms and improved health-related quality of life (HRQoL).

## 3. Materials and Methods

The study was a longitudinal randomized controlled trial utilizing a control and intervention group design. The intervention group received advanced practice nurse (APRN) home-based services over 12 months. The control group received treatment as usual which may included case management. Study enrollment began in September 2004 and ended in April 2008. All study participants provided written, informed consent. The study was approved by the University of Pennsylvania Institutional Review Board and by the City of Philadelphia Health Institutional Review Board. 

### 3.1. Sample

Participants were included in the study if they (1) were age 18 or older, (2) spoke English, (3) lived within the city limits of Philadelphia, (4) had a physician diagnosed SMI, and (5) were HIV positive. They were randomly assigned to treatment as usual (control group) or to the intervention group. The sample was recruited by advertisements placed in mental health and HIV treatment facilities. Participants could self-refer as being HIV positive and receiving treatment for SMI. Following informed consent, all participants received a standard HIV screen at baseline to confirm seropositive status. Any participant not receiving treatment for HIV was referred to the Infectious Disease Outpatient Clinic at the Hospital of the University of Pennsylvania. All participants were paid $40 for each of four interviews over the 12-month study period, as well as for one 24-month followup. A bonus of $100 was paid to participants who provided data at all five study time points. Eligible consenting participants were randomly assigned on a 1 : 1 basis to the intervention and control groups. Randomization ensured that approximately equal numbers of patients were assigned to each of the two groups, which were balanced with respect to observed and unmeasured baseline factors. 

Research assistants (RAs) screened and enrolled participants after obtaining their informed consent. Once these processes were completed, the RAs notified the project manager, who assigned participants to study groups by using a computer-generated algorithm for randomization. Subsequently, the project director notified the APRNs when a participant was assigned to the intervention group. Baseline, 3-, 6-, and 12-month data were collected from both groups by the RAs, who were blinded to study group assignments and hypotheses. The RAs conducted interviews independent from the delivery of nursing services.

### 3.2. Study Intervention

Participants in the intervention group were assigned an APRN who cared for them over the 52 weeks of the study. The APRNs had a Master's degree in nursing; they had a mean of 16.5 years experience in psychiatric mental health nursing (range 4–30 years). By protocol, the APRNs were to meet weekly in a face-to-face contact with the participant. However, phone contact was the alternative when an appointment could not be scheduled. At the first contact, the APRN obtained a full health assessment, including general medical, mental, and environmental health. A plan of care was established in collaboration with the client with a focus on maximizing the participant's ability to self-care. The goal of the program was to improve client outcomes by clarifying, coordinating, and managing treatment regimens and addressing individual and system barriers to care. The APRNs worked closely with each client's case manager, boarding homes, shelters, pharmacies, and clinical providers. APRNs worked toward consistent and reliable information among the various providers by attending appointments with the client and, with the client's permission, sharing updates in treatment regimens, such as medication changes and changes in mental and general health status. APRNs advocated for the client with providers and coached clients to interact more effectively with their providers.

### 3.3. Measures

Changes in psychiatric symptoms and HRQoL over the 12 months of the intervention were the study outcomes. HRQoL was measured with the Medical Outcomes Study 12-Item Short-Form Health Survey (SF-12); psychiatric symptoms were measured with the Patient Health Questionnaire (PHQ-9) and the Colorado Symptom Index (CSI). 

The PHQ-9 is a self-administered screen for depressive symptoms [22]. The PHQ-9 uses the criteria for depression from the fourth edition of the *Diagnostic and Statistical Manual for Mental Disorders*. Each of the nine items is rated on a Likert scale from 0 (*not at all*) to 3 (*nearly every day*). Scores range from 0 to 27. A score of 10–14 indicates mild to moderate depression, 15–19 indicates moderately severe depression, and ≥20 indicates severe depression [22]. The PHQ-9 is a widely used instrument. In 2006, the Centers for Disease Control and Prevention and the Center for Mental Health Services at the Substance Abuse and Mental Health Services Administration began using the PHQ-9 for state-level tracking of outcomes. Forty-one states and territories in 2006 and 16 states in 2008 used the PHQ-9 for outcome benchmarking [23]. 

Psychiatric symptoms were assessed with the CSI, the only psychiatric symptomatology measure developed specifically for community-living persons with mental illnesses. The CSI is a brief, 14-item self-report scale that measures psychiatric symptoms an individual has experienced during the past month, including anxiety, depression, psychotic symptoms, and disturbed thought process [24, 25]. Responses are made on a 5-point scale that ranges from *at least every day* to *not at all*. The internal consistency of the instrument is high for the SMI population (*α* = .89) [24]. A CSI score >30 indicates moderate to severe illness. 

Health-related quality of life (HRQoL) was measured with the SF-12, which assesses eight health domains: physical functioning, role-physical, bodily pain, general health, vitality, social functioning, role activities and mental health. The SF-12 is based on 12 items taken from the SF-36 Health Survey, a standardized questionnaire used to assess patient health. The SF-12 is widely used in clinical trials and routine outcome assessment because of its brevity and psychometric performance. All SF-12 items are scored so a higher value indicates a better health state (0–100) [26]. All scores above or below 50 can be interpreted as above or below the general population norm. Norm-based scoring algorithms are used in this study and based on 1998 SF-36 U.S. population norms [27]. 

Demographic variables included age, gender, race, marital status, employment, income, and living situation.

### 3.4. Nurse Dose

The nurse dose was defined as a combination of three components: the time, the intensity of the need (contact, mode, and setting of the communication by the APRN), and the duration of the APRN intervention. The nurse dose measure was developed and validated using an expert panel of nurse researchers. For analysis in this study, the nurse dose was aggregated to four time points: baseline to 3 months, 3 months to 6 months, 6 months to 12 months, and baseline to 12 months. Daily logs were kept by APRNs to collect detailed data on time, service provided, and communication (contact, mode, and setting).

**Time and Type of Service**

Time was defined as the time it took for the APRN to perform a service. The type of service was defined using the Omaha System (OS) intervention schema [28]. The OS intervention schema includes four service types: Teaching Guidance and Counseling, Treatment and Procedures, Case Management, and Surveillance. An APRN could provide all or any of these services in a given day. Time was assigned to each service then summed for the day.

**Intensity of Need**

It was defined as a composite score of three categories: (1) contact: the person to whom the intervention was directed (client, provider, or other), (2) the Mode of communication (face-to-face, telephone, or other), and (3) the Setting where the intervention was delivered (home, office, or other). Each intensity category was assigned a discrete number that reflected an increasing magnitude of need (e.g., other = 1, provider = 3, client = 5). The following assumptions were used to assign an intensity to the three categories: (a) contact: the client is the most intense focus of a service; next is a provider, and the least intense are other persons, (b) mode: face-to-face service is more intense than telephone contact and telephone contact is more intense than other forms of communication such as e-mail; other is the least intense form of communication, and (c) setting: the client's home is the most intense place for delivering a service, the office is second and other is the least intense. For example, if the nurse provided a face-to-face intervention for the client in their home, the total intensity of need score would be equal to 15, the highest possible intensity. Intensity of need was assigned to each service. A composite intensity of need score was calculated daily.

**Duration**

The duration of the APRN intervention was defined as the total number of weeks the client was in communication with the APRN. As noted above, the study protocol prescribed 52 weeks of the APRN intervention. The duration calculation was the actual number of weeks of the 52 weeks that the APRN was in communication with the participant.

A nurse dose was calculated for each participant in the intervention group at 3, 6, and 12 months. Steps for calculating the nurse dose included the following: (1) time and an intensity score was calculated at the daily level and then summed for 3, 6, and 12 months and (2) the time and intensity score at each time point was divided by the duration (weeks) to yield the nurse dose for that time period.

For ease of analysis and interpretation, we created a categorical APRN dose variable for each participant in the intervention group that reflected a low, moderate, or high dose at each of the four time panels (baseline to 3 months, 3 months to 6 months, 6 months to 12 months, and baseline to 12 months). This categorization was done by ranking all continuous dose quantities in the intervention group across all time periods and then assigning the first tertile as 1, the second as 2, and the top tertile as 3. Participants in the control group were assigned a nurse dose of 0.

### 3.5. Statistical Analysis

Analysis included descriptive measures and intent-to-treat modelling procedures. Baseline characteristics were tested for differences between control and experimental groups with *t* tests for normally distributed continuous variables and with Wilcoxon ranked-sum tests for abnormally distributed variables. Maximum likelihood chi-square was used for categorical variables. In keeping with the intent-to-treat principle, participants who did not complete the study were used in the analyses. Because of participants' nonadherence to treatment, the intent-to-treat analyses likely underestimated the true efficacy of an intervention. However, the intent-to-treat analyses accurately estimate effectiveness for any population in which nonadherence history is similar to that of the intent-to-treat sample. 

We first ran an analysis of the relative differences in change between the intervention and control groups for our measurable outcomes, using a repeated measures random regression model and the time and group interaction term in PROC MIXED of SAS 9.2 (SAS Institute, Cary, NC) to characterize the longitudinal differences between the intervention and control arms. We took PHQ-9, CSI, and SF-12 scores as our outcome measures and derived average treatment effects (ATEs) for each outcome at each of four time panels: baseline to 3 months, 3 months to 6 months, 6 months to 12 months, and baseline to 12 months. We chose to use a random regression model because it allowed us to examine the differences in change in the magnitude of nurse dose over time, or the ATE. The implementation of this model also permitted us to conduct intent-to-treat analyses that included participants with missing outcome data at any time panel under the missing-at-random assumption.

After running our group analysis, we then further examined the differences between the control arm and the three nurse dose subgroups of the intervention arm. Nurse dose was computed for each intervention participant at the four time panels and categorized as low dose, moderate dose, and high dose. We were interested in quantifying the effect of dose magnitude on changes over time in psychiatric symptomatology and health-related quality of life at each time panel. Specifically, we tested for the effect of dose on CSI, PHQ-9, and SF-12 scores during each time panel by using the repeated measures random regression used in the group analysis. For each of the outcome measures, we used the Dose Level × Time interaction in the model to calculate the ATE.

## 4. Results and Discussion

### 4.1. Demographic and Clinical Characteristics

A total of 238 HIV-positive participants with SMI were enrolled in the study, of which 128 participants were randomly assigned to the intervention group and 110 participants were assigned to the control group. From the intervention group, 3 participants were lost to death and 4 to incarceration; an additional 4 participants formally withdrew from the study, and 2 were found ineligible after the randomization process. From the control group, 5 participants were lost to followup because of death.

Table 1 shows the patient characteristics of our sample. The experimental and control groups were similar in all sociodemographic and baseline health characteristics. Diagnoses of mental disorders included schizophrenia spectrum disorders; specifically, schizophrenia, paranoia, delusional disorders, psychosis not otherwise specified, and schizoaffective disorder. Affective disorders were the most common and included major depression, bipolar disorders, and anxiety disorders. Other SMI included borderline personality disorders, substance use, acute reaction to stress, and impulse disorder.

### 4.2. Group-Outcome Analysis

Table 2 shows the ATE estimates between the experimental and control groups for psychiatric symptoms from the PHQ-9 and CSI. Participants from both groups experienced decreases in CSI score from baseline to 12 months, but the relative difference in these improvements was not significant (*d* = −4.03, *P* = .51 (−15.99, 7.83)). During the same period, we found that PHQ-9 scores in control group decreased (*d* = −1.23, *P* = .054 (−2.48, 0.020)) compared to an overall increase for the experimental group (*d* = 3.17, *P* = .37 (−3.78, 10.11]), resulting in an ATE of an increase in PHQ-9 score of 4.40 (*P* = .222 (−2.66, 11.46)).

With regard to the health-related quality-of-life outcomes (e.g., SF-12 mental health score), we found that the Group × Time interactions in our repeated measured random regression models were all nonsignificant (*P* > .05), suggesting no clear difference in the changes in these measurable outcomes over time between the intervention and control groups. The ATEs for the four time panels did not show any significant trends for any of the quality-of-life variables. The analysis is available on request.

### 4.3. Dose-Outcome Analysis

**Figure 1:**
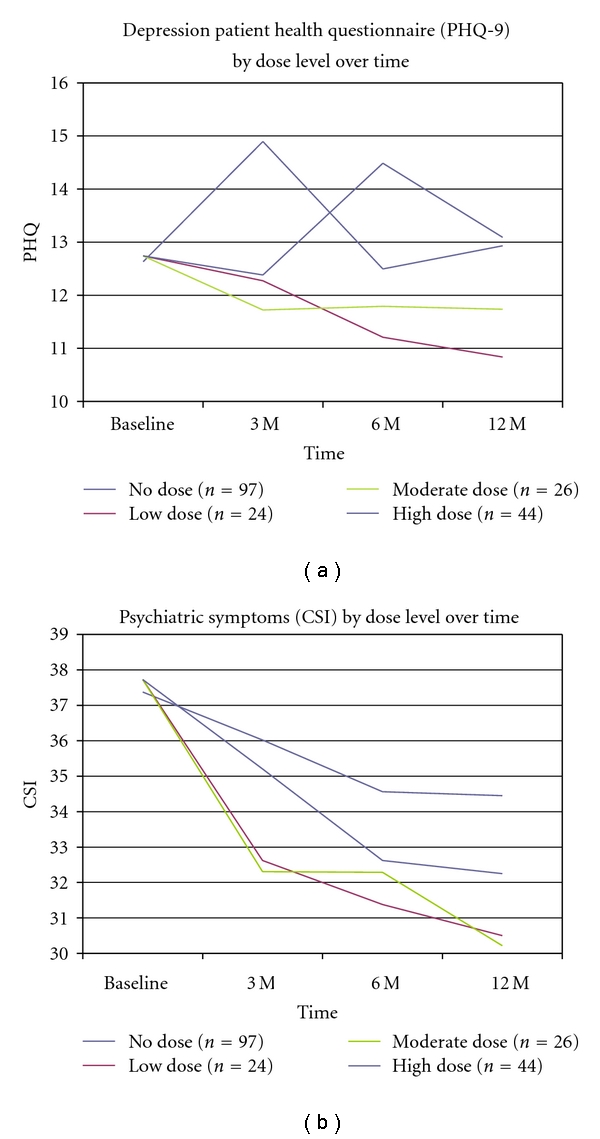


After assigning each intervention participant at each time period a nurse dose level of low, moderate, or high, we then compared the three dose groups with the control group.  Figure 1 depicts the progression of the psychiatric symptom outcomes by dose level. The dose-specific trends suggest heterogeneity in the effect of the APRN intervention among experimental participants; that is, outcome response may have been a function of dose level, rather than only of treatment group. 

Table 3 outlines the regression results for the psychiatric and depression outcomes. We found that the magnitude and direction of the reduction of psychiatric symptomatology, as captured by the CSI score, was most consistent for participants receiving a high dose of APRN intervention. High-dose participants showed a reduction in CSI score at each of the four time panels, and we found that the reductions in CSI scores for these participants were greater than the changes in the usual care group at each time point. In particular, the ATE for the 6- to 12-month period was −5.63 (*P* = .05 (−11.2, −0.01)), and −3.69 from baseline to 12 months (*P* = .102 (−8.1, 0.70)). In contrast, participants receiving a moderate dose had three negative ATEs; from 3 to 6 months, CSI scores of usual care participants decreased more than for participants receiving a moderate APRN dose (ATE = 0.49, *P* = .80 (−3.3, 4.3)). For participants in the low-dose category, we found no distinguishable differences in reduction of CSI scores, compared with those in the usual care group. Namely, the CSI scores in the usual care group decreased more than the CSI scores among the low-dose participants during two of the four time panels. Comparisons of reductions in PHQ-9 scores among varying dose levels followed a trend similar to that of CSI score. From baseline to 12 months, participants in the high-dose category experienced an average decrease in PHQ-9 score of 5.314 points (*P* < .01 (−7.4, 3.16)), compared with a decrease of 1.148 points for the usual care group (*P* = .114 (−2.57, 0.27)), for an ATE of −4.17 (*P* = .002 (−6.7,  −1.6)). During the same period with usual care as the reference group, the ATE was −0.40 (*P* = .79 (−3.4, 2.6)) for moderate-dose participants and −1.07 (*P* = .502 (−4.12, 2.1)) for those receiving a low dose.

Table 4 summarizes the ATEs for the health-related quality outcomes. There appeared to be a strong relationship between APRN dose level and health-related quality-of-life outcome, as suggested by the significant time and nurse dose interaction terms in the regression models. For example, we found that participants in the high-dose group improved their mental health score from baseline to 12 months 0.56 units more than did the usual care group (*P* = .01), compared with an ATE for the moderate group of 0.27 units (*P* = .27). Further, using general health as our summary measure, we observed an ATE of 0.55 units from baseline to 12 months for the high-dose group (*P* = .01), compared with −0.02 for the moderate group (*P* = .95), and −0.19 for the low-dose group (*P* = .47).

## 5. Discussion

This study of the effectiveness of a care management intervention by APRNs for patients with SMI and HIV demonstrated improvement in symptoms and health-related quality of life. The ATE showed significant reductions in symptoms for the intervention group at the higher nurse dose level but not at the low or moderate nurse dose level. These results suggest that care management by APRNs may be a useful strategy for improving care and outcomes for high need individuals with SMI and HIV. The APRNs facilitated improvement through a combination of education, medication management, and advocacy within the health system. These findings are consistent with a growing body of literature that suggests that care management models are beneficial for vulnerable populations. People with SMI and HIV share with these populations high risk for adverse outcomes due to complex medical and psychiatric profiles and complicated treatment regimens. 

 Components of the APRN care management intervention make it an appealing approach for improving treatment outcomes among community-dwelling individuals with SMI. APRNs have the specialty education that integrates mental and general health care. The system-level barriers are often insurmountable for individuals with SMI. Care is delayed or not obtained at all, and the illness continues to worsen, often developing into a full-blown crisis needing high-end and expensive care in emergency departments or hospitalization. Compared with usual care, care management by APRNs may be a more efficient approach for the high-risk SMI and HIV population. The APRN provides patient-centered care by delivering services in the client's home environment. Additionally, the client has quick access to advanced assessment and surveillance of an APRN. Studies using APRNs versus registered nurses show the APRN to be more effective in meeting the needs of high-risk populations because they have the authority to manage the health care needs in the moment without the delay of referral to a physician [29]. APRNs are independently licensed in most states to prescribe and treat health conditions. Improving accessibility to health care might be associated with mental health improvement over the long term by lowering stress level. Future studies need to evaluate health biomarkers such as cardiometabolic markers over the long term to establish overall improvement in health in this population [30]. 

In both the intervention and control groups, participants had high PHQ-9 and CSI scores indicating a moderate to high level of psychiatric symptoms when compared with the general public. In addition, the participants scored consistently lower on the health-related quality-of-life survey than the general population. Many other studies have shown similar refractory patterns in symptoms with the SMI population [30]. Changes or improvement in conditions are difficult to detect. In our study, the APRN dose-response analysis showed patterns not revealed in standard group analyses. For example, heterogeneity in the effect of the APRN intervention was discovered among the intervention participants, indicating that the outcome response may be a function of dose level, rather than only treatment group. From another point of view, the dose-effect pattern shows there are individuals who may require higher doses of the APRN to achieve improvement. 

Figure 1 demonstrates patterns of response to the APRN dose. Compared with the control group, it appears that the intervention group who had low and moderate APRN dose had lower depression scores (PHQ-9) in all 12 months of the study. The high APRN dose receivers showed a different pattern. Depression appeared to improve in the first 3 months then worsened in the 3 to 6 month time period, then improved again in the last 6 months of the study.  Figure 1 also shows patterns in psychiatric symptoms (CSI). All levels of the APRN dose recipients show greater improvement than the controls. Most important is the observation that there are different patterns among those in the intervention group. High-dose participants experienced a reduction in CSI score of 3–5 points at each of the four time panels, and we found that the reductions in the CSI score were greater for participants in the intervention group than for those in the usual care group at each time point. Other than noting the direction of the change and differences among the dose recipients and the control group, we are reluctant to draw conclusions. Future studies are required to benchmark the response to APRN interventions. 

As health care reform is implemented, an opportunity presents itself to ensure that system changes are made in the provision of care for complex patient populations, such as those with HIV and SMI. In the current health care system, the SMI patient with HIV infection would most likely be referred to an HIV medical provider in a location separate from his or her mental health care, requiring the patient to be responsible for arranging and keeping the appointment as well as finding transportation. This fragmented system does not promote optimal outcomes for the HIV-SMI population. The concept of the “health home” that promotes collaborative care among specialties could be translated into APRN-led treatment centers in the community that provide cost-effective and quality care specifically to this population. In this sense, the “home” for receiving health care services (physical and mental health) could be a virtual home centered on a home care model.

We note some limitations to our study. The APRNs used in this study were university based and had training in research. Therefore, results may or may not be different from community-based nurses. Although the control group was not given the nursing intervention, the repeated interviewing by the research assistants at the multiple time points could be considered a form of “intervention.” The idea is supported by the fact that the control group experienced improvements similar to the intervention group. The addition of a third APRN without the intended reduction of randomized assignments to the first two nurses resulted in unequal randomization probabilities and an imbalance in sample sizes across treatment groups and the three nurses. A fourth nurse was used as a replacement for the first two nurses for a subset of patients. These changes may have inserted an indirect bias into the study.

## 6. Conclusions

This study demonstrated that people with SMI and HIV could achieve improvement with APRN care management services. This population is a high need, high cost subgroup with poor quality of overall health. The personal and societal costs of these problems are staggering. Implementation of community-based nurse management using APRNs for complex patient populations may improve long-term outcomes and reduce the high costs of care. This study suggests that APRN care management should be a central component of the redesign of health care delivery to this vulnerable population.

## Figures and Tables

**Table 1:** Baseline characteristics of sample receiving an intervention from an advanced practice psychiatric nurse or usual care.

Characteristic	APRN intervention (*n* = 128)		Control (*n* = 110)		*P*
	*n*	%	*n*	%	
Age (mean ± SD)	43.9 ± 6.6		43.2 ± 7.7		.42
Gender					.98
Male	67	52.3	58	52.7	
Female	57	44.5	49	44.5	
Transgender	4	3.1	3	2.7	
Race or ethnicity					.47
Black or African American	105	81.9	88	79.1	
White	13	10.2	11	10	
American Indian	2	1.6	2	1.8	
More than one race	0	—	3	2.7	
Other	8	6.3	6	6.4	
Hispanic or Latino	13	10.2	8	7.3	.58
Education					.68
Less than high school	66	51.9	52	47.3	
High school	36	27.8	38	34.5	
Post-high school technical training	2	1.6	1	0.9	
Some college	16	12.3	16	14.5	
College degree	5	4	2	1.9	
Graduate studies	3	2.4	1	0.9	
Current employment status					.30
Unemployed	114	89.1	97	88.2	
Competitive job	8	6.3	7	6.4	
Transitional employment	2	1.6	1	0.9	
Work training	2	1.6	0	—	
Work in sheltered workshop	1	0.8	0	—	
Other	1	0.8	5	4.5	
Mental illness					.51
Schizophrenia spectrum disorder	25	19.7	28	25.7	
Affective disorder	94	73.2	76	68.8	
Other serious mental illness	9	7.1	6	5.5	
Years from HIV diagnosis to					
Baseline interview date (mean ± SD)	11.8 ± 5.7		12.4 ± 6.5		.77

**Table 2:** Average treatment effects for patients receiving an advanced practice psychiatric nurse intervention.

Period	Depression (PHQ-9)^b^			Psychiatric symptoms (CSI)^c^		
	Effect (*d*)	95% CI	*P* ^ a^	Effect (*d*)	95% CI	*P* ^a^
Baseline to 3 months	2.99	−4.01, 9.99	.402	−2.58	−14.35, 9.19	.667
3 months to 6 months	2.39	0.53, 4.24	.012	−0.32	−3.44, 2.79	.841
6 months to 12 months	−0.98	−2.97, 1.02	.336	−1.14	−4.46, 2.19	.503
Baseline to 12 months	4.40	−2.66, 11.46	.222	−4.03	−15.99, 7.83	.505

^a^Compared with participants who received usual care.

^b^Patient Health Questionnaire (PHQ-9).

^c^Colorado Symptom Index (CSI).

**Table 3:** Depression and psychiatric symptom outcomes: average treatment effects for patients receiving an advanced practice psychiatric nurse intervention, by dose versus usual care.

APRN dose	Period	Depression (PHQ-9)^a^			Psychiatric symptoms (CSI)^b^		
		Effect (*d*)	95% CI	*P* ^c^	Effect (*d*)	95% CI	*P* ^c^
Low	Baseline to 3 M	−2.35	−*5.1, 0.4 *	.09	−1.50	−*5.8, 2.8 *	.50
	3 M to 6 M	1.81	−*0.6, 4.0 *	.14	0.73	−*3.3, 4.8 *	.73
	6 M to 12 M	−0.69	−*3.3, 1.9 *	.60	0.69	−*3.5, 4.9 *	.75
	Baseline to 12 M	−1.07	−*4.2, 2.1 *	.50	−2.11	−*7.4, 3.2 *	.44

Moderate	Baseline to 3 M	−1.50	−*4.2, 1.2 *	.27	−3.28	−*7.6, 1.0 *	.14
	3 M to 6 M	2.39	*0.2, 4.6*	**.03**	0.49	−*3.3, 4.3 *	.80
	6 M to 12 M	−1.90	−*4.9, 1.1 *	.22	−2.23	−*7.1, 2.6 *	.37
	Baseline to 12 M	−0.40	−*3.4, 2.6 *	.79	−4.40	−*9.4, 0.6 *	.09

High	Baseline to 3 M	−4.19	−*6.4, –2.0 *	**<.001**	−0.74	−*4.2, 2.7 *	.68
	3 M to 6 M	2.26	−*0.9, 5.4 *	.16	−3.11	−*8.5, 2.3 *	.26
	6 M to 12 M	−1.60	−*5.1, 1.9 *	.36	−5.63	−*11.2, *−*0.01 *	**.05**
	Baseline to 12 M	−4.17	−*6.7, –1.6 *	**.00**	−3.69	−*8.1, 0.7 *	.10

Note. Negative effect values indicate a decrease in symptoms and improvement.

^a^Patient health questionnaire (Colorado symptom Index).

^b^Colorado symptom Index.

^c^Compared with participants who received usual care.

**Table 4:** Health-related quality outcomes: average treatment effects for patients receiving advanced practice registered nurse intervention, by dose, versus usual care.

APRN dose	Period	Effect (*d*)	95% CI	*P*	Effect (*d*)	95% CI	*P*	Effect (*d*)	95% CI	*P*	Effect (d)	95% CI	*P*	Effect (*d*)	95% CI	*P*	
		Mental composite			Physical Composite			Mental health			General health			Social functioning			
Low	Baseline to 3 M	1.25	−*1.7, 4.2 *	.42	−0.55	−*3.7, 2.6 *	.73	0.19	−*0.2, 0.6 *	.40	−0.01	−*0.4, 0.4 *	.94	−0.01	−*0.5, 0.5 *	.94	
	3 M to 6 M	0.59	−*2.5, 3.6 *	.70	−1.75	−*4.9, 1.4 *	.27	0.01	−*0.4, 0.4 *	.95	−0.10	−*0.6, 0.4 *	.67	−0.12	−*0.7, 0.4 *	.65	
	6 M to 12 M	0.84	−*2.5, 4.1 *	.61	0.44	−*3.1, 3.9 *	.80	0.19	−*0.2, 0.6 *	.43	−0.15	−*0.6, 0.4 *	.55	0.01	−*0.5, 0.6 *	.97	
	Baseline to 12 M	2.99	−*0.2, 6.2 *	.07	−3.60	−*7.3, 0.1 *	.05	0.56	*0.4, 0.6*	.76	−0.19	−*0.7, 0.3 *	.47	0.12	−*0.5, 0.7 *	.70	

Moderate	Baseline to 3 M	0.57	−*2.4, 3.5 *	.71	−0.11	−*3.3, 3.0 *	.94	0.01	−*0.4, 0.4 *	.98	0.15	−*0.3, 0.6 *	.50	0.03	−*0.5, 0.5 *	.94	
	3 M to 6 M	0.90	−*1.9, 3.7 *	.53	−1.12	− *4.0, 1.8*	.45	−0.13	−*0.5, 0.3 *	.54	−0.07	−*0.5, 0.4 *	.75	−0.04	−*0.5, 0.4 *	.88	
	6 M to 12 M	1.46	−*2.4, 5.3 *	.47	0.75	−*3.3, 4.8 *	.71	0.27	−*0.3, 0.8 *	.35	−0.12	−*0.7, 0.5 *	.68	0.05	−*0.6, 0.7 *	.88	
	Baseline to 12 M	2.23	−*0.8, 5.3 *	.15	0.16	−*3.3, 3.6 *	.92	0.27	−*0.2, 0.7 *	.27	−0.02	−*0.5, 0.5 *	.95	−0.01	−*0.6, 0.5 *	.94	

High	Baseline to 3 M	−0.57	−*2.9, 1.8 *	.64	1.90	−*0.6, 4.4 *	.14	0.29	−*0.1, 0.6 *	.12	0.45	*0.1, 0.8*	.02	−0.13	−*0.6, 0.3 *	.54	
	3 M to 6 M	5.55	*1.4, 9.6*	.01	−4.14	−*8.3, 0.03 *	.05	0.46	−*0.1, 1.0 *	.13	0.06	−*0.6, 0.7 *	.85	0.35	−*0.4, 1.1 *	.34	
	6 M to 12 M	1.46	−*2.8, 5.8 *	.50	2.90	−*1.7, 7.5 *	.21	0.27	−*0.3, 0.9 *	.39	−0.01	−*07, 0.7 *	.97	−0.21	−*0.9, 0.5 *	.57	
	Baseline to 12 M	1.87	−*0.8, 4.5 *	.17	2.60	−*0.4, 5.6 *	.09	0.56	*0.1, 0.9*	.01	0.55	*0.1, 1.0*	.01	−0.37	−*0.9, 0.1 *	.16	

		Vitality			Role activities			Physical role			Bodily pain			Physical functioning			

Low	Baseline to 3 M	0.10	−*0.3, 0.5 *	.65	−0.02	−*0.5, 0.4 *	.93	−0.15	−*0.6, 0.3 *	.55	0.04	−*0.3, 0.4 *	.82	0.12	−*0.2, 0.4 *	.52	
	3 M to 6 M	0.03	−*0.4, 0.4 *	.87	0.03	−*0.4, 0.4 *	.86	−0.02	−*0.6, 0.5 *	.93	−0.12	−*0.4, 0.2 *	.51	−0.26	−*0.6, 0.1 *	.14	
	6 M to 12 M	0.02	−*0.4, 0.4 *	.92	0.12	−*0.2, 0.5 *	.52	0.17	−*0.3, 0.7 *	.49	0.18	−*0.2, 0.5 *	.37	0.04	−*0.3, 0.4 *	.83	
	Baseline to 12 M	0.06	−*0.4, 0.5 *	.79	0.21	−*0.3, 0.7 *	.40	−0.38	−*0.9, 0.1 *	.15	0.00	−*0.4, 0.4 *	.98	−0.27	−*0.7, 0.1 *	.16	

Moderate	Baseline to 3 M	−0.12	−*0.6, 0.3 *	.59	0.17	−*0.3, 0.6 *	.46	−0.11	−*0.6, 0.4 *	.65	0.08	−*0.3, 0.4 *	.68	0.03	−*0.3, 0.4 *	.85	
	3 M to 6 M	0.15	−*0.2, 0.5 *	.42	0.20	−*0.1, 0.5 *	.23	−0.08	−*0.6, 0.4 *	.74	−0.16	−*0.5, 0.1 *	.35	−0.02	−*0.3, 0.3 *	.93	
	6 M to 12 M	−0.09	−*0.6, 0.4 *	.73	0.23	−*0.2, 0.7 *	.29	0.03	−*0.5, 0.6 *	.92	0.42	−*0.04, 0.9 *	.07	0.19	−*0.3, 0.6 *	.40	
	Baseline to 12 M	−0.16	−*0.6, 0.3 *	.45	0.48	*0.03, 0.9*	.04	0.20	−*0.3, 0.7 *	.43	0.38	−*0.05, 0.8 *	.08	−0.08	−*0.4, 0.3 *	.66	

High	Baseline to 3 M	−0.26	−*0.6, 0.1 *	.15	−0.14	−*0.5, 0.2 *	.46	−0.05	−*0.4, 0.3 *	.79	0.35	*0.02, 0.6*	.03	0.10	−*0.2, 0.4 *	.46	
	3 M to 6 M	−0.35	−*0.9, 0.2 *	.19	0.51	*0.04, 0.9*	.03	0.01	−*0.7, 0.7 *	.99	−0.35	−*0.8, 0.1 *	.14	−0.31	−*0.8, 0.2 *	.18	
	6 M to 12 M	0.06	−*0.5, 0.6 *	.83	0.63	*0.1, 1.1*	.01	0.37	−*0.3, 1.0 *	.25	0.65	*0.1, 1.1*	.01	0.30	−*0.2, 0.8 *	.22	
	Baseline to 12 M	−0.36	−*0.7,* −*0.01 *	.05	0.57	*0.2, 0.9*	.01	0.24	−*0.2, 0.7 *	.27	0.57	*0.18, 0.9*	.00	−0.23	−*0.1, 0.5 *	.15	

Note: Health-related quality of life was measured with the medical outcomes study 12-item short form health survey. Positive effect values indicate improvement.
